# Efficient Delivery of Therapeutic siRNA by Fe_3_O_4_ Magnetic Nanoparticles into Oral Cancer Cells

**DOI:** 10.3390/pharmaceutics11110615

**Published:** 2019-11-17

**Authors:** Lili Jin, Qiuyu Wang, Jiayu Chen, Zixiang Wang, Hongchuan Xin, Dianbao Zhang

**Affiliations:** 1 School of Life Science, Liaoning University, Shenyang 110036, China; lilijin@lnu.edu.cn (L.J.); qiuyuwang@lnu.edu.cn (Q.W.); zxwlnu@163.com (Z.W.); 2 Department of Stem Cells and Regenerative Medicine, Key Laboratory of Cell Biology, National Health Commission of China, and Key Laboratory of Medical Cell Biology, Ministry of Education of China, China Medical University, Shenyang 110122, China; chenjiayu@cmu.edu.cn; 3 Qingdao Institute of Bioenergy and Bioprocess Technology, Chinese Academy of Sciences, Qingdao 266101, China; xinhc@qibebt.ac.cn

**Keywords:** magnetic nanoparticle, iron oxide, siRNA delivery, BCL2, BIRC5/survivin, oral cancer

## Abstract

The incidence of oral cancer is increasing due to smoking, drinking, and human papillomavirus (HPV) infection, while the current treatments are not satisfactory. Small interfering RNA (siRNA)-based therapy has brought hope, but an efficient delivery system is still needed. Here, polyethyleneimine (PEI)-modified magnetic Fe_3_O_4_ nanoparticles were prepared for the delivery of therapeutic siRNAs targeting B-cell lymphoma-2 (BCL2) and Baculoviral IAP repeat-containing 5 (BIRC5) into Ca9-22 oral cancer cells. The cationic nanoparticles were characterized by transmission electronic microscopy (TEM), scanning electronic microscopy (SEM), dynamic light scattering (DLS), and vibrating sample magnetometer (VSM). By gel retardation assay, the nanoparticles were found to block siRNA in a concentration-dependent manner. The cellular uptake of the nanoparticle/siRNA complexes under a magnetic field was visualized by Perl’s Prussian blue staining and FAM labeling. High gene silencing efficiencies were determined by quantitative real-time PCR and western blotting. Furthermore, the nanoparticle-delivered siRNAs targeting BCL2 and BIRC5 were found to remarkably inhibit the viability and migration of Ca9-22 cells, by cell counting kit-8 assay and transwell assay. In this study, we have developed a novel siRNA-based therapeutic strategy targeting BCL2 and BIRC5 for oral cancer.

## 1. Introduction

The incidence of oral cancer has increased due to risk factors such as tobacco, alcohol, and human papillomavirus (HPV), resulting in nearly 180 thousand deaths worldwide in 2018 [1,2]. The clinical treatment of oral cancer mainly depends on surgery, radiotherapy, chemotherapy, and several targeted drugs, but the prognosis is poor [3,4]. Therefore, it is necessary to develop novel therapeutic strategies to overcome the limitations of current therapies for oral cancer.

Recent progress in nanotechnology-based gene therapy has brought hopes for cancer treatment [5]. RNA interference (RNAi) is a sequence-specific post-transcriptional gene silencing process in eukaryotes [6]. RNAi could be triggered by microRNA (miRNA) and small interfering RNA (siRNA), which could be designed to target almost any gene [7]. It is exploited by researchers for loss-of-function studies and holds promise for the development of therapeutic gene silencing [8]. The first siRNA drug Onpattro (patisiran) targeting transthyretin (TTR) has been approved by the U.S. Food and Drug Administration (FDA) in 2018, for the treatment of peripheral nerve disease polyneuropathy in adults. This major progress, with the elucidation of more and more disease-related target genes, has greatly stimulated the research and development of siRNA drugs. However, how to effectively deliver siRNA drugs is a bottleneck to clinical practice [6,9]. The currently developed siRNA delivery strategies mainly include siRNA conjugation, lipid-based, and polymer-based delivery systems [10,11]. In our previous studies, the prepared polyethyleneimine (PEI)-coated Fe_3_O_4_ nanoparticles exhibited siRNA protection and delivery capacities for mesenchymal stem cells and glioblastoma cells [12,13]. However, the feasibility of these type of nanoparticles for delivering siRNA to oral cancer cells remains unknown, and the uniformity and efficiency of delivery needs to be improved.

An increasing number of cancer target genes has been reported in recent years. BCL2 (B-cell lymphoma-2) is a gene that is overexpressed in many cancers to escape cell death [14]. It is a promising cancer therapeutic target, but there are few targeting agents with clinical significance [14,15,16]. For oral cancers, BCL2 were proved to be important in cancer progression and chemoradiation resistance [17]. Inhibition of BCL2 in oral cancer cells inhibited proliferation and induced apoptosis, and also augmented the inhibitory effects of cisplatin in vitro and in vivo [18]. BIRC5 (Baculoviral IAP repeat-containing 5, also named survivin) is a conserved gene essential for cell proliferation. It is expressed in proliferating cells and upregulated in most cancers, the inhibition of BIRC5 leads to apoptosis or sensitization to chemotherapy and radiotherapy [19,20]. BIRC5 is rarely mutated in oral cancer samples and upregulated compared to non-cancerous tissue [21]. In this study, we prepared Fe_3_O_4_ nanoparticles and design siRNAs targeting BCL2 and BIRC5, aiming to explore the efficient delivery of therapeutic siRNA into oral cancer cells by Fe_3_O_4_ nanoparticles, which might provide a novel strategy for the future therapy of oral cancer.

## 2. Materials and Methods

### 2.1. Synthesis and Characterization of Nanoparticles

The magnetic nanoparticles were synthesized based on the oxidative hydrolysis method [22]. Briefly, FeSO_4_ and PEI dissolved in H_2_SO_4_ solution were dripped into KNO_3_ and NaOH solutions under nitrogen bubbling in a triple neck flask. After precipitation, the PEI–Fe_3_O_4_ nanoparticles were obtained by ultrafiltration (100 KDa, UFC910096, Millipore, Beijing, China). The particle size and morphology were analyzed by transmission electronic microscopy (TEM) and scanning electronic microscopy (SEM) [12]. The particle size was analyzed by ImageJ software. The zeta potential and hydrodynamic size of nanoparticles were analyzed by dynamic light scattering (DLS, Mastersizer 2000, Malvern, Worcestershire, UK). The amino group density was determined by the conductivity meter, and the elemental content was analyzed by energy dispersive spectroscopy (EDS). The magnetization of nanoparticles was measured by the vibrating sample magnetometer (VSM, Lakeshore7404, Westerville, OH, USA).

### 2.2. Gel Retardation Assay

The binding capacity of the nanoparticles to siRNA was analyzed by gel retardation assay. In general, 1 µg siRNA was mixed with 0, 0.5, 1, 1.5, and 2 µg nanoparticles in Opti-MEM Reduced Serum Medium (51985034, Gibco, Shanghai, China) and incubated at room temperature for 10 min. The mixtures were subjected to 2% agarose gel electrophoresis at 100 V for 20 min. The gels were stained with Biosafe nucleic acid dye (170-3001, Tanon, Shanghai, China) and imaged under Tanon-1600 imaging system (Tanon).

### 2.3. Cell Culture

Human oral cancer cell Ca9-22 and CAL 27 (Procell, Wuhan, China) cells were cultured in Dulbecco’s Modified Eagle Medium (DMEM) with high glucose (SH30022.01, Hyclone, Beijing, China) supplemented with 10% Certified Fetal Bovine Serum (FBS) (04-001-1A, Bioind, Kibbutz Beit-Haemek, Israel) and 1% Penicillin Streptomycin (SV30010, Hyclone) at 37 °C in a humidified atmosphere containing 5% CO_2_.

### 2.4. Cell Transfection

The Ca9-22 cells were seeded on 24-well culture plates and incubated for 12 h before transfection. Normally, 0.6 μg of nanoparticles and 0.2 μg of siRNA were diluted with 20 μL of Opti-MEM Reduced Serum Medium separately and then mixed together. The mixtures were incubated at room temperature for 10 min and added to the cells. The cells were incubated under the magnetic field for 30 min (Mag0201, Nanoeast, Nanjing, China) and then under normal conditions. The transfection of siRNA using Lipofectamine 3000 Transfection Reagent (L3000015, Invitrogen, Shanghai, China) was carried out according to the manufacturer’s instructions, serving as a positive control. FAM-siRNAs were used for observation of the cellular uptake of siRNA by fluorescence microscope (Observer A1, Carl Zeiss, Oberkochen, Germany). All the siRNA duplexes were chemically synthesized by GenePharma (Shanghai, China) and the sequences were as follows: siBCL2—5′-GGGAGAACAGGGUACGAUATT-3′; siBIRC5—5′-GAAGCAGUUUGAAGAAUUATT-3′; NC (non-targeting siRNA serving as a negative control) 5′-UCCGAACGUGUCACGUTT-3′.

### 2.5. Perl’s Prussian Blue Staining

The cellular uptake of the nanoparticles was visualized by Perl’s Prussian blue staining. The cells were cultured in 24-well plates and transfected as indicated. After incubation for 12 h, the cells were fixed with 4% Paraformaldehyde Fix Solution (P0099, Beyotime) for 15 min. The cells were washed with PBS and stained with 5% potassium ferrocyanide in 10% hydrochloric acid for 30 min at room temperature. The working solution was made by mixing equal volumes of 10% potassium ferrocyanide and 20% hydrochloric acid solution just before use. The cells were observed under a microscope (Olympus CKX41, Tokyo, Japan).

### 2.6. Quantitative Real-time PCR

The mRNA levels of genes were analyzed by quantitative real-time PCR. Total RNA was prepared using RNAiso Plus (9108, Takara, Dalian, China) according to the manufacturer’s instructions. The RNA concentration and quality were determined by NanoDrop 2000C spectrophotometer (Thermo Fisher Scientific, Wilmington, DE, USA). Equal amounts total RNA from samples were subjected to reverse transcription separately using PrimeScript RT reagent Kit with gDNA Eraser (Perfect Real Time) (RR047A, Takara). The TB Green Premix Ex Taq II (Tli RNaseH Plus) (RR820A, Takara) was used for quantitative real-time PCR in a 7500 Real-Time PCR Systems (Applied Biosystems, Foster City, CA, USA) following the standard protocol provided by the manufacturer. The relative mRNA levels were calculated by the ΔΔCt method and GAPDH served as an internal control. All the primers were chemically synthesized by Sangon Biotech (Shanghai, China). The sequences of primers were listed in Table 1.

### 2.7. Western Blotting

The protein levels were determined by the western blotting assay. Total protein lysis was prepared using the RIPA Lysis Buffer (P0013B, Beyotime) and quantified by the BCA Protein Assay Kit (T9300A, Takara). The protein samples for western blotting were prepared using SDS-PAGE Sample Loading Buffer (P0015L, Beyotime). Equal amounts of total proteins were loaded onto 10% SDS-PAGE (P0012AC, Beyotime) and separated by electrophoresis. The separated proteins were transferred onto a PVDF membrane (IPVH00010, Millipore, Shanghai, China) and blocked by 5% skim milk (232100, BD Bioscience, San Jose, CA, USA). The membranes were incubated with primary antibodies Bcl-2 Rabbit Polyclonal Antibody (1:1000, AF0060, Beyotime), Anti-Survivin Rabbit pAb (1:1000, GB11177, Servicebio, Wuhan, China) and GAPDH Mouse Monoclonal antibody (1:5000, 60004-1-Ig, Proteintech, Wuhan, China) at 4 °C overnight. Then HRP-conjugated Affinipure Goat Anti-Mouse IgG (1:5000, SA00001-1, Proteintech) and HRP-conjugated Affinipure Goat Anti-Rabbit IgG (1:5000, SA00001-2, Proteintech) were used to probe the membrane at room temperature for 1 h. The protein bands were visualized using Amersham ECL Prime Western Blotting Detection Reagent (RPN2232, GE Healthcare, Princeton, NJ, USA) and imaged by Tanon-5200 chemiluminescence detection system (Tanon).

### 2.8. Cell Viability Assay

The cell viability was analyzed using Cell Counting Kit-8 assay (MA0218, Meilunbio, Dalian, China). In brief, the cells were cultured in 24-well plates and transfected as indicated, and then seeded into 96-well plates. A 10 μL of CCK-8 solution was added to each well and incubated at 37 °C for 1 h. The absorbance at 450 nm was detected using an iMARK microplate reader (Bio-Rad, Hercules, CA, USA) with a reference wavelength of 630 nm.

### 2.9. Transwell Migration Assay

The migration capacity of cells was assessed using the transwell migration assay. The CA9-22 cells were transfected as indicated for 24 h and then seeded with serum-free culture medium into the upper chamber of Transwell (3422, Coring, Corning, NY, USA). The complete medium was added into the lower chamber. After incubation for 12 h, the cells were fixed with 4% Paraformaldehyde Fix Solution for 15 min. The cells on the upper side of the membranes were removed with a cotton swab. The migrated cells were stained with Crystal Violet Staining Solution (C0121, Beyotime) and visualized using a microscope (Olympus CKX41).

### 2.10. Cell Cycle Analysis

The cell cycle progression was determined using the Cell Cycle and Apoptosis Analysis Kit (C1052, Beyotime). The cells were collected and fixed in ice-cold 70% ethanol overnight. The fixed cells were washed with PBS and stained with PI in Staining Buffer supplemented with RNase A at 37 °C for 30 min in the dark. Then the stained cells were analyzed by flow cytometry FACSCalibur (BD Bioscience).

### 2.11. Statistical Analysis

The experiments were carried out in triplicates and the data were presented as mean ± standard deviation (SD). Statistical significance was determined by one-way analysis of variance (ANOVA) following post-hoc multiple comparisons. *p* < 0.05 was considered to be statistically significant.

## 3. Results

### 3.1. Nanoparticle Characterization

**Figure 1:**
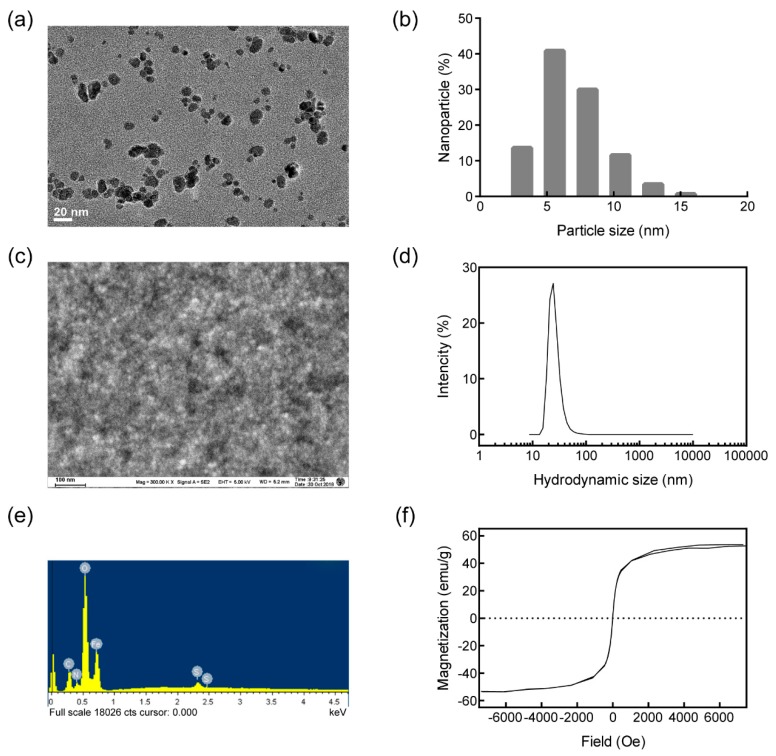
Characterization of the prepared nanoparticles. (**a**) The transmission electron microscope (TEM) image of the nanoparticles, and the bar indicates 20 nm; (**b**) the diameter distribution of the nanoparticles; (**c**) the scanning electron microscopy (SEM) image of the nanoparticles, and the bar indicates 100 nm; (**d**) the distribution of the hydrodynamic diameter of the nanoparticles; (**e**) the element composition; (**f**) the magnetization curve of the nanoparticles.

TEM and SEM were carried out to characterize the prepared nanoparticles. As shown in Figure 1a, the synthesized nanoparticles presented a dense spherical morphology. Based on the TEM image, the diameter of nanoparticles was analyzed and the average diameter was 7.95 nm (Figure 1b). The SEM image in Figure 1c further confirmed the uniform morphology and good dispersion of the nanoparticles. The hydrodynamic diameter of the nanoparticles was detected by DLS, the average diameter was 26.12 nm (Figure 1d). By EDS energy-mapping, the element nitrogen was proven to contribute 10.6% of the dry weight of the nanoparticles, indicating PEI accounted for about 33.6% (Figure 1e). The amino group density analyzed by the conductivity meter was 1482 nmol/g. The average zeta potential analyzed by DLS was +46.5 mV, further quantifying the positive charge of the nanoparticles. The magnetization curve obtained by VSM showed saturation magnetization as 52.7 emu/g, without a hysteresis loop (Figure 1f). These data proved that the prepared Fe_3_O_4_ nanoparticles were small, superparamagnetic, and positively charged.

### 3.2. Cellular Upkake of Nanoparticle/siRNA

**Figure 2:**
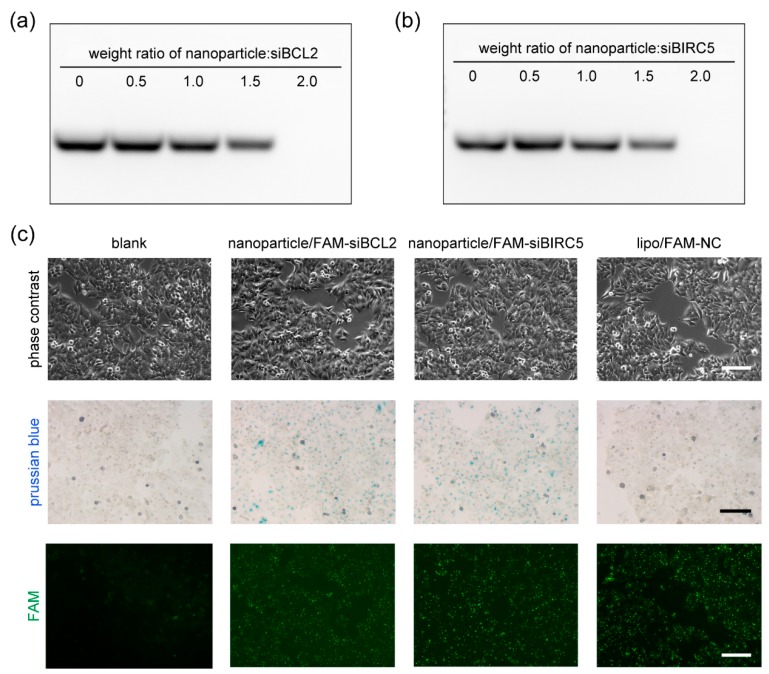
The cellular uptake of the siRNA mediated by the nanoparticles. (**a**) Gel retardation assay for the interaction between nanoparticles and siBCL2; (**b**) gel retardation assay for the interaction between nanoparticles and siBIRC5; (**c**) the delivery of siRNAs into Ca9-22 cells by the nanoparticles visualized by Perl’s Prussian blue staining and FAM-labeled siRNAs. Lipofectamine 3000 (lipo) was served as a positive control for siRNA transfection. Bar indicates 100 μm.

The capacity of nanoparticles to form complexes with siRNA ex vitro is essential for siRNA delivery into cells, and it is also key to optimize the delivery parameters. The gel retardation assay was performed to assess the interaction between the nanoparticles and siRNA. After 1 µg of siRNA was incubated with 0, 0.5, 1, 1.5, and 2 µg of nanoparticles and subjected to agarose gel electrophoresis. When the siRNA formed complexes with the nanoparticles, the complexes were so large that they would not move under the electric field in the agarose gel and remain in the loading holes. The observed bands indicated the free siRNA; it presented concentration-dependent gel retardation in both siBCL2 (Figure 2a) and siBIRC5 (Figure 2b) gel images. When 2 µg of nanoparticles were incubated with 1 µg of siRNA, all the siRNA could not run in the agarose gel electrophoresis, suggesting that siRNA totally formed complexes with nanoparticles when the weight ratio was more than 2.0. In other words, when the weight ratio was less than 2.0, the ability of nanoparticles to load siRNA was saturated or nearly saturated. Most of the positive charges of nanoparticles were neutralized by negatively charged siRNA, it was not conducive for the complexes to approach the surface of cell membranes, which was negatively charged. Thus, more nanoparticles were essential for siRNA delivery into the cells. For siRNA delivery, 0.6 μg of nanoparticles and 0.2 μg of siRNA were used for Ca9-22 cells in each well of 24-well plates. The cells were incubated under the magnetic field for 30 min and then under normal conditions for 12 h. The cellular uptake of the nanoparticle/siRNA was detected by Perl’s Prussian blue staining and FAM-labeled siRNAs. As shown in Figure 2c, almost all the cells were stained blue in nanoparticle-delivered FAM-siBCL2 and siBIRC5 groups. Under a fluorescence microscope, green fluorescence was observed in these two groups and lipo (lipofectamine 3000)/FAM-NC group (positive control). These results indicated an efficient siRNA delivery by the nanoparticles into Ca9-22 cells.

### 3.3. Gene-Silencing Efficiency

**Figure 3:**
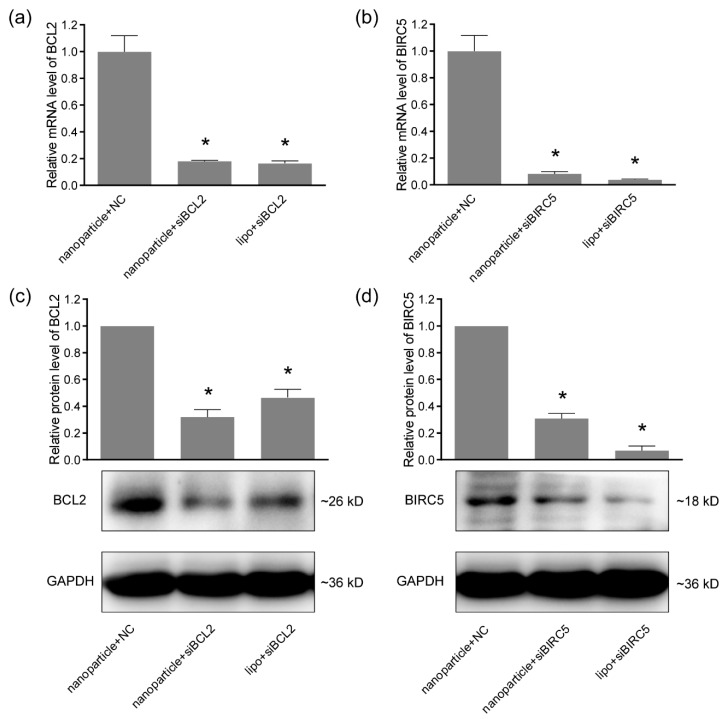
The gene silencing efficiencies of siRNA delivered by nanoparticles in Ca9-22 cells. (**a**) The mRNA levels of BCL2 in Ca9-22 cells detected by quantitative real-time PCR; (**b**) the mRNA levels of BIRC5 detected by quantitative real-time PCR; (**c**) the protein levels of BCL2 analyzed by western blotting; (**d**) the protein levels of BIRC5 analyzed by western blotting. GAPDH was served as an internal control. Lipofectamine 3000 (lipo) was served as a positive control for siRNA transfection. * *p* < 0.05 compared with nanoparticle+NC (negative control) group.

**Figure 4:**
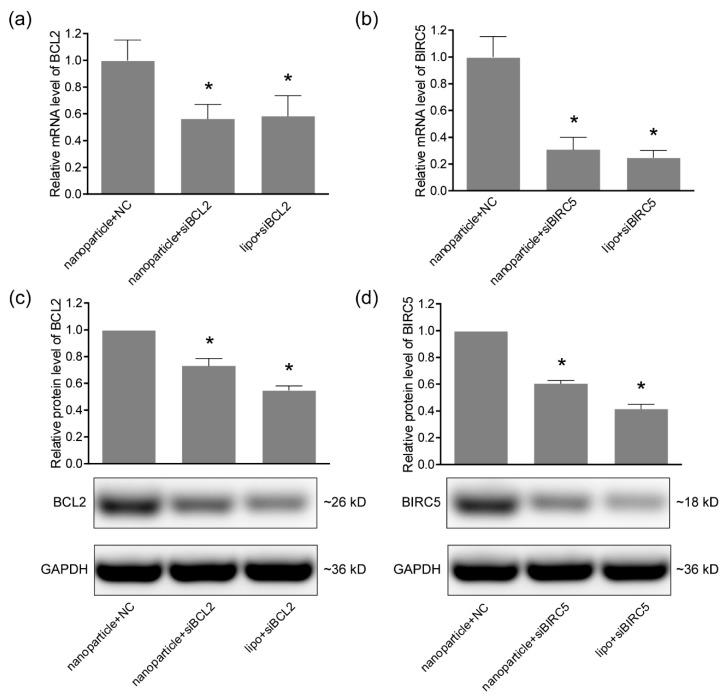
The gene silencing efficiencies of siRNA delivered by nanoparticles in CAL 27 cells. (**a**) The mRNA levels of BCL2 in CAL 27 cells detected by quantitative real-time PCR; (**b**) the mRNA levels of BIRC5 detected by quantitative real-time PCR; (**c**) the protein levels of BCL2 analyzed by western blotting; (**d**) the protein levels of BIRC5 analyzed by western blotting. GAPDH was served as an internal control. Lipofectamine 3000 (lipo) was served as a positive control for siRNA transfection. * *p* < 0.05 compared with nanoparticle+NC (negative control) group.

To determine the gene-silencing efficiency of nanoparticle-delivered siRNA in oral cancer cells, 0.6 μg of nanoparticles and 0.2 μg of siRNA were used for Ca9-22 and CAL 27 cells in each well of 24-well plates, the cells were incubated under the magnetic field for 30 min and then under normal conditions for 48 h. Total RNA was extracted and analyzed using quantitative real-time PCR. As shown in Figure 3a and Figure 4a, the mRNA level of BCL2 was significantly reduced by nanoparticle-delivered siBCL2 to 18% in Ca9-22 cells and 56% in CAL 27 cells, compared with the nanoparticle+NC groups. The silencing of BCL2 was further verified in the protein level using western blotting (Figure 3c and Figure 4c). For BIRC5 silencing, similar results were observed in Figure 3b,d and Figure 4b,d. The BIRC5 in Ca9-22 was significantly silenced by nanoparticle-delivered siBIRC5 and proven by quantitative real-time PCR and western blotting in both mRNA and protein levels. Lipofectamine 3000 (lipo) was served as a positive control for siRNA transfection. These data proved the satisfied siRNA delivery efficiency of the nanoparticles in Ca9-22 and CAL 27 cells, at least for BCL2 and BIRC5 silencing.

### 3.4. Anti-Tumor Activity

**Figure 5:**
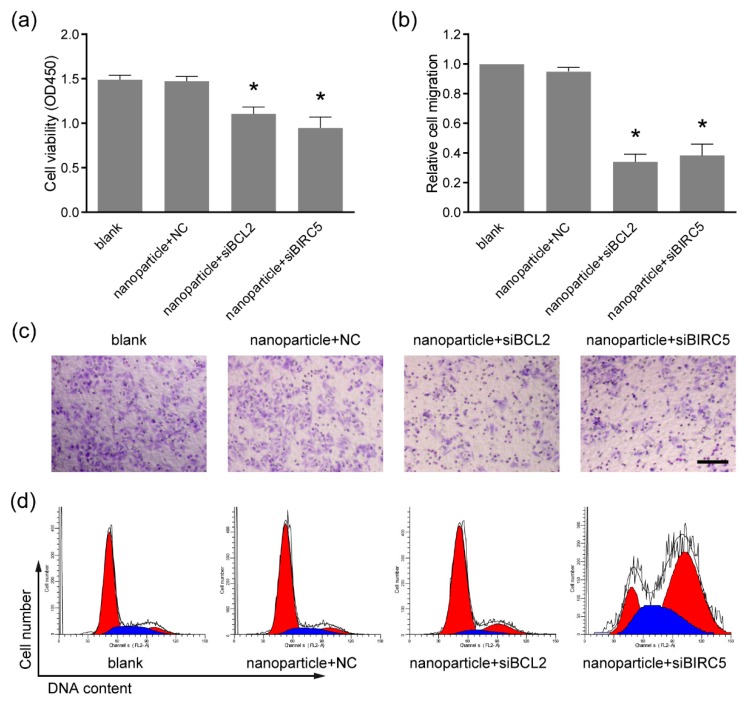
Cell viability and migration were inhibited by the siRNA delivered by nanoparticles. (**a**) The cell viability examined by cell counting kit-8 assay (CCK-8). * *p* < 0.05 compared with nanoparticle+NC group; (**b**) the cell migration analyzed by the transwell assay; (**c**) the representative images of migrated cells in the transwell assay. Bar indicates 100 μm. (**d**) The cell cycle distribution analyzed by PI staining followed by flow cytometry. G1 phase arrest and G2 phase arrest were observed upon nanoparticle-delivered siBCL2 and siBIRC5, respectively.

To evaluate the anti-tumor activity of the nanoparticle-delivered therapeutic siRNA targeting BCL2 and BIRC5, the siRNAs were delivered to Ca9-22 cells by the nanoparticles as described in Section 3.3. By the CCK-8 assay, cell viability showed significant reduction in the nanoparticle+siBCL2 and nanoparticle+siBIRC5 groups, compared with the nanoparticle+NC group (Figure 5a). The treatment with nanoparticle+NC also showed no significant difference versus blank groups, indicating the safety of the nanoparticles at working concentrations (0.6 μg per well of 24-well plate). Our previous data showed that these types of nanoparticles were toxic to cells at high concentrations [23]. Thus, the nanoparticles need to be used at appropriate concentrations to avoid cytotoxicity. The migration capacity of tumor cells is critical for cancer metastasis [24], and the Ca9-22 cell migration was determined using the transwell assay. As shown in Figure 5b,c, the numbers of migrated cells were remarkably reduced by the nanoparticle-delivered siBCL2 and siBIRC5. Furthermore, cell cycle distribution was analyzed by flow cytometry to verify the inhibitory effects. G1 phase cell cycle arrest was observed in nanoparticle+siBCL2 group and G2 phase arrest in nanoparticle+siBIRC5 group (Figure 5d). These results demonstrated the effective delivery of therapeutic siRNA to Ca9-22 cells by the nanoparticles, indicating a novel potential therapeutic strategy for oral cancer therapy.

## 4. Discussion

Investigations for molecular mechanisms during oral cancer occurrence and development provide a large number of candidate target genes, but most of which have not been targeted for therapy until now [4]. RNAi-based gene therapy expands the scope of targeted therapies, due to rational drug design instead of high-throughput screening and the increased target space including non-druggable targets and non-coding RNA [5,9]. Meanwhile, the stability, bioavailability, and the delivery across biomembranes still limit siRNA drug development [25,26,27,28]. In previous studies, cationic polymer polyethylenimine-modified nanoparticles were used for siRNA delivery to oral cancer cells. PEI-modified mesoporous silica nanoparticles were used for codelivery of doxorubicin and MDR1-siRNA to overcome multidrug resistance for oral squamous carcinoma treatment [29]. Polyethylene glycol-polyethyleneimine-chlorin e6 (PEG-PEI-Ce6) nanoparticles delivered Wnt-1 siRNA to the cytoplasm of KB cells enhanced the cancer cell-killing effect by photodynamic therapy (PDT) [30]. Here, a type of PEI-modified Fe_3_O_4_ nanoparticle was prepared for siRNA delivery into Ca9-22 oral cancer cells. The zeta potential of the cationic nanoparticle was +46.5 mV, providing satisfied siRNA adsorption capacity and dispersibility, as verified by SEM, TEM, and gel retardation. The gel retardation data indicated that the siRNA targeting BCL2 and BIRC5 could be completely blocked by the nanoparticles of twice siRNA mass, achieved by the high PEI content and amino group density. During siRNA delivery, the PEI coating on the Fe_3_O_4_ core provided a positive charge, which is essential for siRNA capture and cellular uptake. For siRNA delivery into Ca9-22 cells, the weight ratio of the nanoparticles to the siRNAs was 3:1 to retain the positive charge of the particles, thereby facilitating delivery into the cells. To enhance the delivery efficiency further, the superparamagnetism of the nanoparticles was utilized by applying an external magnetic field when transfection. As presented in the images obtained by Prussian blue staining and FAM labeling, cellular uptake of the nanoparticles and siRNAs was uniform with high-efficiency for both siBCL2 and siBIRC5, which was comparable with commercial in vitro transfection regent Lipofectamine 3000. The superparamagnetic property could be utilized for magnetofection and MIR imaging in future studies.

The siRNA adsorbed by nanoparticles needs to be released after entering cells to play the role of gene silencing by forming an RNA-induced silencing complex (RISC). To verify the effectiveness of the nanoparticle-delivered siRNA in oral cancer cells, designed siRNAs targeting BCL2 and siBIRC5 were used separately. The gene silencing efficiency was verified by quantitative real-time PCR and western blotting at mRNA and protein levels. Both siBCL2 and siBIRC5 achieved high silencing potencies, indicating the efficient siRNA delivery by the Fe_3_O_4_ nanoparticles into Ca9-22 cells. While the silencing efficiency was lower in oral cancer CAL 27 cells. The gene silencing efficacy of RNAi relies on siRNA design, delivery strategy, and cell properties [31,32,33]. The same siRNA sequences and delivery methods were used for the two cell lines. The positive control group treated with lipo also showed lower silencing efficiency in CAL 27 cells. Therefore, it was speculated that CAL 27 cells might be more difficult to receive transfection or the activation of the RNAi pathway was lower, leading to the lower efficiency of gene silencing. These phenomena drive us to further optimize the delivery scheme and improve the structure of nanoparticles in future research in more cells and in vivo experiments. In order to further verify the anti-cancer effects of the delivered siRNAs, cell viabilities and migration were proved to be remarkably inhibited by either siBCL2 or siBIRC5. The molecular weight and charge of the synthesized siRNAs are similar, so the delivery system based on the Fe_3_O_4_ nanoparticles would be suitable for more reasonably designed siRNA delivery and would not be limited to cancer therapy. In summary, we have successfully developed a novel therapeutic strategy for oral cancer, as well as a simple and universal siRNA delivery system for more applications.

## Figures and Tables

**Table 1:** Primer sequences for quantitative real-time PCR.

Gene	Primer Sequence	Product Length (bp)
*BCL2*	forward: 5′-GATAACGGAGGCTGGGATGC-3′	105
	reverse: 5′-CAGGGCCAAACTGAGCAGAG-3′	
*BIRC5*	forward: 5′-TGAGAACGAGCCAGACTTGG-3′	86
	reverse: 5′-GTTCCTCTATGGGGTCGTCA-3′	
*GAPDH* [13]	forward: 5′-GCACCGTCAAGGCTGAGAAC-3′	138
	reverse: 5′-TGGTGAAGACGCCAGTGGA-3′	
